# ARTEMISININ BIOSYNTHESIS PROMOTING KINASE 1 positively regulates artemisinin biosynthesis through phosphorylating AabZIP1

**DOI:** 10.1093/jxb/erx444

**Published:** 2017-12-28

**Authors:** Fangyuan Zhang, Lien Xiang, Qin Yu, Haoxing Zhang, Taixin Zhang, Junlan Zeng, Chen Geng, Ling Li, Xueqing Fu, Qian Shen, Chunxian Yang, Xiaozhong Lan, Min Chen, Kexuan Tang, Zhihua Liao

**Affiliations:** 1 Key Laboratory of Eco-environments in Three Gorges Reservoir Region (Ministry of Education), Chongqing Key Laboratory of Plant Ecology and Resources Research in Three Gorges Reservoir Region, SWU-TAAHC Medicinal Plant Joint R&D Centre, School of Life Sciences, Southwest University, Chongqing, China; 2 College of Life Sciences and Oceanography, Shenzhen University, Shenzhen, China; 3 Joint International Research Laboratory of Metabolic & Developmental Sciences, Key Laboratory of Urban Agriculture (South) Ministry of Agriculture, Plant Biotechnology Research Center, School of Agriculture and Biology, Shanghai Jiao Tong University, Shanghai, China; 4 TAAHC-SWU Medicinal Plant Joint R&D Centre, Tibet Agricultural and Husbandry College, Nyingchi of Tibet, China; 5 College of Pharmaceutical Sciences, Key Laboratory of Luminescent and Real-Time Analytical Chemistry (Ministry of Education), Southwest University, Chongqing, China

**Keywords:** *Artemisia annua*, artemisinin biosynthesis, bZIP1, kinase, phosphorylation, transgene

## Abstract

The plant *Artemisia annua* produces the anti-malarial compound artemisinin. Although the transcriptional regulation of artemisinin biosynthesis has been extensively studied, its post-translational regulatory mechanisms, especially that of protein phosphorylation, remain unknown. Here, we report that an ABA-responsive kinase (AaAPK1), a member of the SnRK2 family, is involved in regulating artemisinin biosynthesis. The physical interaction of AaAPK1 with AabZIP1 was confirmed by multiple assays, including yeast two-hybrid, bimolecular fluorescence complementation, and pull-down. *AaAPK1*, mainly expressed in flower buds and leaves, could be induced by ABA, drought, and NaCl treatments. Phos-tag mobility shift assays indicated that AaAPK1 phosphorylated both itself and AabZIP1. As a result, the phosphorylated AaAPK1 significantly enhanced the transactivational activity of AabZIP1 on the artemisinin biosynthesis genes. Substituting the Ser^37^ with Ala^37^ of AabZIP1 significantly suppressed its phosphorylation, which inhibited the transactivational activity of AabZIP1. Consistent overexpression of *AaAPK1* significantly increased the production of artemisinin, as well as the expression levels of the artemisinin biosynthesis genes. Our study opens a window into the regulatory network underlying artemisinin biosynthesis at the post-translational level. Importantly, and for the first time, we provide evidence for why the kinase gene *AaAPK1* is a key candidate for the metabolic engineering of artemisinin biosynthesis.

## Teaser

An ABA-responsive SnRK2-type kinase, AaAPK1, is involved in positively regulating artemisinin biosynthesis in *Artemisia annua* through phosphorylation of AabZIP1.

## Introduction

Malaria is a serious and sometimes fatal disease that put nearly half of the world’s population at risk in 2015 (World Health Organization, 2015). Artemisinin-based combination therapies (ACTs) are recommended by the World Health Organization (WHO) for the cure of malaria (World Health Organization, 2015). The Chinese herbal plant, *Artemisia annua*, is the only commercially available resource for artemisinin production (Peplow, 2016); however, the artemisinin content is very low in the plants and this has led to a global shortage in its supply for malarial treatment (Tang *et al.*, 2014). Recently, artemisinin has also been shown to produce promising therapeutic effects for diabetes (Li *et al.*, 2017) and tuberculosis (Zheng *et al.*, 2017), and these discoveries are further increasing the global demand for artemisinin and its derivatives. There is urgent pressure to develop new varieties of *A*. *annua* with high yields of artemisinin, and metabolic engineering approaches provide a proven effective strategy to do this. Artemisinin biosynthesis has been well studied at the molecular and biochemical levels, and several artemisinin biosynthesis genes, such as *ADS*, *CYP71AV1*, and *DBR2*, have been successfully used to improve production in transgenic plants of *A*. *annua* (Tang *et al.*, 2014). Having elucidated the process of artemisinin biosynthesis, plant scientists are now paying more attention to, and making great progress in, determining its transcriptional regulation.

A series of transcription factors have been identified in the artemisinin biosynthesis pathway. Most of them, including *AaWRKY1* (Ma *et al.*, 2009), *AaERF1/2* (Yu *et al.*, 2012), *AaORA* (Lu *et al.*, 2013), *AaTAR1* (Tan *et al.*, 2015), *AaHD1* (Yan *et al.*, 2017), and *AabHLH1* (Ji *et al.* 2014), have been found to be responsive to methyl jasmonate (MeJA). For example, *AaMYC2* (Shen *et al.*, 2016*a*) and *AaGSW1* (Chen *et al.*, 2017) are up-regulated by MeJA and abscisic acid (ABA), and *AaNAC1* is inducible by salicylic acid (SA) and MeJA (Lv *et al.*, 2016). Among the transcription factors, *AabZIP1* is the only one that is specifically up-regulated by ABA (Zhang *et al.*, 2015). AabZIP1 positively regulates artemisinin biosynthesis by directly binding to the promoters of *ADS* and *CYP71AV1* (Zhang *et al.*, 2015). The crosstalk between MeJA and ABA signaling in artemisinin production has provided a means to uncover the regulatory network of its biosynthesis (Shen *et al.*, 2016*b*). Recently, a fine feed-forward loop model has been proposed (Chen *et al.*, 2017). At the transcriptional level, both the MeJA-responsive AaMYC2 and the ABA-responsive AabZIP1 positively regulate the trichome-specific expression of AaGSW1 by binding to its promoter, thus suggesting that AaGSW1 integrates the signals of MeJA and ABA to govern artemisinin biosynthesis and that it positively and directly regulates *AaORA* and *CYP71AV1* (Chen *et al.*, 2017). At the post-translational level, JAZ proteins repress the activity of AaMYC2 and AaHD1 through physical interactions (Yan *et al.*, 2017). However, the positive regulators of artemisinin biosynthesis at the post-translational level, such as the related kinases that are involved, have not yet been identified.

Phosphorylation of transcription factors plays an important role in regulating metabolite biosynthesis at the post-translational level. Camalexin is one of the primary defense metabolites *in planta*, and MPK3/MPK6 regulate its production via direct phosphorylation of WRKY33, which positively regulates camalexin biosynthesis by directly binding to the promoter of *CYP71B15* in *Arabidopsis thaliana* (Mao *et al.*, 2011). The R2R3 MYB transcription factor MYB75 regulates anthocyanin biosynthesis and its phosphorylation by MAP KINASE 4 (MPK4) is required for a fully functional MYB75 (Li *et al.*, 2016). In *Catharansus roseus*, the MAP kinase cascade, composed of CrMAPKKK1, CrMAPKK1, and CrMAPK3/6, regulates terpenoid indole alkaloid (TIA) biosynthesis by phosphorylating CrMYC2 and ORCAs (Paul *et al.*, 2017). In Arabidopsis, some bZIP-type transcription factors, namely AREBs/ABFs, act as positive regulators in the ABA signaling pathway (Yoshida *et al.*, 2010). The activation of AREBs requires an ABA-dependent post-transcriptional modification, such as that of phosphorylation by sucrose nonfermenting-1 (Snf1)-related protein kinases (SnRK2s; Yang *et al.*, 2017). To date, however, the role of transcription-factor phosphorylation in the regulation of metabolite biosynthesis, including that of artemisinin, has not been well studied and is poorly understood.

Previously, we have characterized an ABA-responsive transcription factor, AabZIP1, that positively regulates artemisinin biosynthesis through its direct binding to the promoters of target genes (Zhang *et al.*, 2015). In this follow-up study, we further identify an SnRK2-type kinase (ARTEMISININ BIOSYNTHESIS PROMOTING KINASE 1, abbreviated as AaAPK1), that regulates artemisinin biosynthesis in *A*. *annua* plants through the phosphorylation of AabZIP1. We provide a comprehensive characterization of AaAPK1 at the molecular, biochemical, and metabolic engineering levels, and highlight its role in promoting the biosynthesis of artemisinin via AabZIP1-phosphorylation in *A. annua*.

## Materials and methods

### Plant materials and chemicals

The Huhao 1 cultivar of *Artemisia annua*, developed in Shanghai (China), was used in this study (Shen *et al.*, 2016*a*; Yan *et al.*, 2017). Seedlings were grown in a greenhouse with a 16-h light photoperiod at 25 ± 1 °C for 5 months, then switched to an 8-h light photoperiod to promote their flowering. Once the plants were flowering, their roots, stems, leaves, and flower buds were separately harvested to analyse gene expression. For the abiotic stresses and ABA treatment, 1-month-old plants were treated with 300 mM NaCl and 10 mM ABA, respectively, then sampled at 0, 3, 6, and 12 h, using the methods reported by Zhang *et al.* (2015). Drought stress was applied by removing the soil from intact 1-month-old plants and leaving them in the air without water supply for 0, 3, 6, or 12 h.

Seeds of *Nicotiana benthamiana* (tobacco) were sown directly into soil and grown under a 16-h light photoperiod at 25 ± 1 °C. Plants at 5 weeks old were used for subcellular localization, bimolecular fluorescence complementation (BiFC), and dual luciferase (Dual-LUC) assays. ABA was purchased from Sigma-Aldrich (St. Louis, USA), while the other chemicals were purchased from the China National Medicines Corporation (Beijing, China).

### Gene discovery and bioinformatics analysis

To search for the SnRK2 subfamily in *A*. *annua*, hidden Markov model (HMM) profiles of SnRK2 were built by HMMER v.3.1 (www.hmmer.org) using the amino acid sequences of SnRK2 subfamily members from Arabidopsis downloaded from TAIR (www.arabidopsis.org). SnRK2 homologs were identified through a HMM search against the *A*. *annua* protein sequence database with an *E* value <10^−9^. The redundant sequences were then manually removed. The protein sequences of Arabidopsis, rice, and *A*. *annua* were aligned using ClustalW (Thompson *et al.*, 1994). A non-rooted phylogenetic tree was constructed by using the maximum-likelihood method, in the MEGA software v.5 (Tamura *et al.*, 2011). For this, a bootstrap analysis was performed with 1000 replicates to evaluate the accuracy of the phylogenetic construction.

### Cloning of the candidate kinases

First-strand cDNA was synthesized with 2 µg of total RNAs that were isolated from the young leaves of *A*. *annua*, and used as the reverse transcriptase PCR templates. PCR was performed following the manufacturer’s instructions for the KOD DNA polymerase (Toyobo, Osaka, Japan). The PCR program was same as described previously (see Zhang *et al.*, 2015). The PCR products were subcloned into the pJET2.1 vector (Thermo Fisher Scientific, Waltham, USA) and sequenced. All the primers used in the study are listed in Supplementary Table S1 at *JXB* online.

### Yeast two-hybrid assays

To perform yeast two-hybrid (Y2H) analysis, the PCR-amplified coding sequences of the candidate kinase genes were each cloned into the pGBKT7 vector (used as the bait plasmid). The coding sequence of AabZIP1 was amplified by PCR and cloned into the pGADT7 vector (used as the prey plasmid). The construct pairs were transformed into a yeast strain, AH109, by using the LiAc method following the instructions for the yeast transformation system (Takara, Japan). Next, the transformed yeasts were selected on synthetic defined SD/–Trp/–Leu (–TL) agar medium plates and cultured at 30 °C. Five individual transformants were randomly chosen, dropped into 10^9^ dilutions on SD/–Trp/–Leu/–His (–TLH), with 5 mM 3-aminotriazole (3-AT) and SD/–Trp/–Leu/–His/–Ade (–TLHA), and allowed to grow for 2–3 d at 30 °C.

### BiFC assays

The gateway-compatible plasmids used in the BiFC analysis were described in Shen *et al.* (2016*a*). The coding sequences of AaAPK1and AabZIP1 were each cloned into the gateway entry vector TOPO-pEntry, following the instructions provided (Invitrogen, Carlsbad, CA, USA). Then, AaAPK1 was fused in-frame with the nYFP-tag of the pEarleygate201-YFP^N^ by the LR recombination reaction, and likewise AabZIP1 was fused in-frame with the cYFP-tag of the pEarleygate202-YFP^C^. The BiFC experiment was performed according to the protocol reported by Schweiger and Schwenkert., (2014). Briefly, the constructs were transformed into an *Agrobacterium tumefaciens* strain, GV3101, and transiently co-transformed into 5-week-old *N*. *benthamiana* leaves. After 48 h of cultivation, the yellow fluorescent protein (YFP) signals were viewed under low-light conditions with confocal microscopy (Olympus, Tokyo, Japan). The pEarleyGate202-YFP^C^ empty vectors served as the controls.

### Subcellular localization analysis

The coding sequence of AaAPK1 was amplified by PCR and cloned into the plant overexpression vector PHB (Zhang *et al.*, 2015): it was positioned in-frame with YFP to generate the PHB-AaAPK1-YFP plasmid. Subsequently, the PHB-AaAPK1-YFP plasmid was transferred into the *A*. *tumefaciens* strain GV3101 and transiently co-transformed into the *N*. *benthamiana* leaves. After 48 h of cultivation, the YFP signals were viewed as described above.

### GST pull-down assay

To express and purify the GST-AabZIP1 protein and AaAPK1-HIS^6^ from *Escherichia coli*, the coding sequence of *AabZIP1* was inserted into the *Bam*HI and *Xho*I sites of pGEX-6P-1, and likewise that of *AaAPK1* into those of pET28a. The plasmids were transformed into the *E*. *coli* strain Rosetta (DE3) for protein expression. Isopropylthio-β-galactoside (IPTG), with a final concentration of 0.1 mM, was added to the medium for the protein induction. The GST-AabZIP1 fusion protein was purified with Glutathione Sepharose 4B (AmershamPharmacia, Little Chalfont, UK). The GST protein was also expressed by using empty pGEX-6P-1 plasmids and purified, as described above. The AaAPK1-HIS^6^ fusion protein was purified with Ni-sepharose (AmershamPharmacia, Little Chalfont, UK). GST-AtABF2 and AtOST1-HIS^6^ were expressed and purified in the same way as for GST-AbZIP1 and AaAPK1-HIS^6^, respectively.

The GST pull-down assay was performed by adding 5 µg of GST or the GST-AabZIP1 fusion protein, 40 µl of Glutathione Sepharose 4B, and 5µg of AaAPK1-HIS^6^ protein into the Eppendorf tubes, and supplementing it with a binding buffer [10 mM of Na_2_HPO_4_, 2 mM of KH_2_PO_4_, 140 mM of NaCl, 2.7 mM of KCl, and 10% (v/v) glycerol] to a final volume of 400 µl. Samples were mixed gently, rotated at 70 rpm for 4 h at 4 °C, and then centrifuged at 500 *g* for 3 min at 4 °C. The supernatant was discarded, and the resins were washed five times with pre-cooled binding buffer. After the final wash, the supernatant was completely discarded, and 30 µl of 1×SDS loading buffer was added. Then, the samples were separated by 10% (w/v) SDS-PAGE and transferred to a PVDF membrane (Amersham Pharmacia, Little Chalfont, UK). The primary antibody was anti-HIS and the secondary antibody was goat anti-mouse antibody (Amersham Pharmacia, Little Chalfont, UK). To detect the proteins, we used an ECL PLUS kit (Pierce, Waltham, MA, USA).

### Phosphorylation assay

To perform the AaAPK1 autophosphorylation assay, 3 µg of AaAPK1-HIS^6^ or the AtOST1-HIS^6^ protein was added into kinase assay buffer (20 mM Tris-HCl buffer, 100mMNaCl, 20 mM MgCl2, 2 mM DTT, with or without 10 mM of ATP) to a final volume of 25 µl. To analyse the phosphorylation of AabZIP1 by AaAPK1, 3 µg of the GST-AabZIP1 protein and 1 µg of the AaAPK1-HIS^6^ protein were simultaneously added to the kinase assay buffer to a final volume of 25 µl. Serving as the positive control, 3 µg GST-AtABF2 and 1 µg AtOST1-HIS^6^ were added to the same kinase assay buffer, to a final volume of 25 µl. For the alkaline phosphatase (AP) treatment, 1 µl of fast alkaline phosphatase (Thermo Fisher, Waltham, MA, USA) was added into the 25 µl kinase assay buffer. Samples were mixed gently and stored at 30 °C for 30 min. Samples were subsequently separated using 8% (w/v) SDS-PAGE, with 0.1 mM of MnCl_2_ and 0.1 mM of Phos-tag (Wako, Tokyo, Japan), and the proteins were detected by Coomassie Blue staining.

### Dual luciferase assay

Dual luciferase (dual-LUC) assays were performed according to previously reported methods (Zhang *et al.*, 2015; Shen *et al.*, 2016*a*). Briefly, the coding sequence of *AaAPK1* was amplified by PCR and cloned into the *Pst*I and *Xba*I sites of the PHB plasmid, to generate the PHB-AaAPK1 construct used as the effector. To serve as the control, YFP was cloned into the PHB plasmid. The promoters of *ADS*, *CYP71AV1*, *DBR2*, and *ALDH1* were inserted into pGREEN-0800 (Hellens *et al.*, 2005) to drive the luciferase reporters. The effector and control constructs were transferred into the *A*. *tumefaciens* strain GV3101. The reporter constructs were co-transformed with pSoup19 into GV3101. Infiltration and detection were done following the protocols described in Zhang *et al.* (2015).

### Establishing transgenic plants of *A*. *annua*

To construct the RNA interference plasmid, a fragment containing a partial open reading frame (ORF) and 3′UTR was cloned into the gateway entry clone, pEntry; this was inserted into the plant RNA interference plasmid pHELLSGATE12 vector via the LR reaction (Invitrogen, Waltham, MA, USA) to generate pHELLSGATE-iAaAPK1. The plant overexpression vector was PHB-AaAPK1 (constructed as described above). The plant overexpression construct, PHB-AaAPK1, and the RNA interference construct, pHELLSGATE-iAaAPK1, were each introduced into the *A*. *tumefaciens* strain EHA105, and subsequently used to genetically transform *A*. *annua* following our previously described methods (Zhang *et al.*, 2013).

### Molecular analysis of the genes of interest

To detect the transgenes, genomic DNA was extracted from the *A*. *annua* plants using our previously reported methods (Zhang *et al.*, 2015). The primers used are listed in Supplementary Table S1. Real-time quantitative PCR (RT-qPCR) analysis was done following previously reported methods (Zhang *et al.*, 2015). Roots, stems, leaves, and flower buds were collected from 5-month-old plants and immediately frozen in liquid nitrogen. For the plants treated with ABA, NaCl, and drought (and their respective controls) only the leaves were collected. To analyse the expression levels of *AaAPK1* and the artemisinin biosynthesis genes, leaves from 2-month-old transgenic and wild-type plants were harvested. The total RNA exacted from these samples was prepared as described above using the total plant RNA Extract Kit (Tiangen, Beijing, China). The relative expression levels of genes were normalized to the expression of *A*. *annua ACTIN*. Real-time PCR was performed in a Bio-Rad MJ thermocycler IQ5 (Bio-Rad, Hercules, USA). Amplification was carried out with the Brilliant SYBR Green QPCR MasterMix (Takara, Japan), according to the manufacturer’s instructions. The thermal profile for SYBR Green real-time PCR was 95 °C for 2 min, followed by 40 cycles of 95 °C for 15 s, and 60 °C for 1 min.

### Detection of artemisinin metabolites

All green leaves were harvested from each 2-month-old *A*. *annua* plant grown in the greenhouse under a 16-h light photoperiod at 25 ± 1 °C. The leaves were dried at 50 °C for 48 h. A 0.2-g sample of the dried leaf powder was extracted with 20 ml of petroleum ether in an ultrasonic bath for 30 min, then filtered and evaporated to dryness in a vacuum at 50 °C. Finally, the residue was re-dissolved in 5 ml of methanol. From the ensuing solution, 1 ml was passed through a 0.22-µm nylon membrane filter. The filtered solutions were then analysed using a Shimadzu LC-6AD HPLC system (Shimadzu ELSD-LTII detector). The HPLC conditions consisted of a Phenomenex C18 column that used an acetonitrile:water (60:40 v/v) mixture as the mobile phase at a flow rate of 1 ml min^−1^. The ELSD conditions were optimized at a nebulizer-gas pressure of 320 kPa and a drift tube temperature of 60 °C, with the gain set to 8. Authentic samples of artemisinin were bought from Sigma and used as the control in the measurements. Each sample had an injection volume of 20 µl.

### Statistics

All experiments that need statistical analysis were performed using at least three biological replicates. Data are given as means ±SD. To compare group differences, paired two-tailed Student’s *t*-tests were used. *P*-values <0.05 were regarded as statistically significance.

## Results

### Cloning and bioinformatic analysis of the candidate kinase genes of *A*. *annua*

**Fig. 1.**
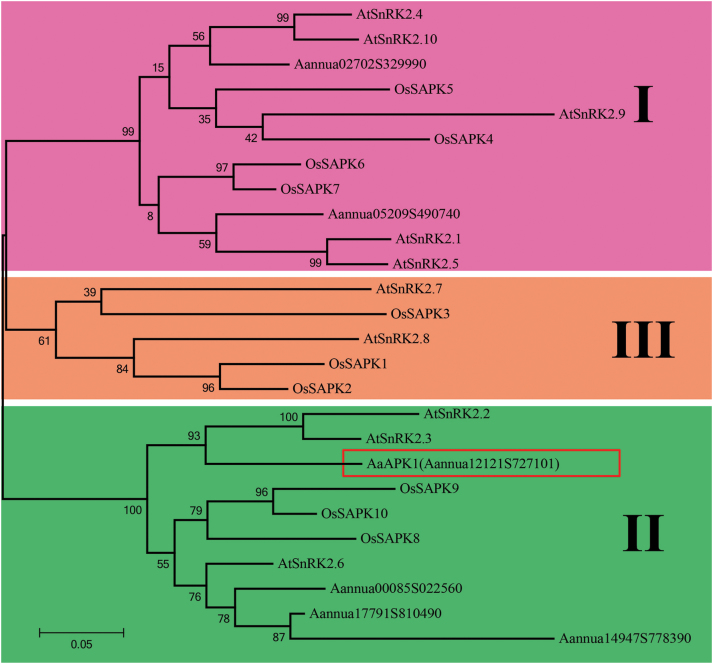
Phylogeny of the SnRK2 subfamily kinase. The unrooted maximum-likelihood tree built with SnRK2s from *Artemisia annua* (Aa), *Arabidopsis thaliana* (At), and *Oryza sativa* (Os). The numbers on the branches showed the bootstrap values (given by 1000 repeats). The scale 0.05 represents the genetic distance. (This figure is available in colour at *JXB* online.)

Recently, we showed that AabZIP1 plays an important functional role in the ABA-induced over-production of artemisinin (Zhang *et al.*, 2015). AabZIP1 is a transcription activator that works by binding to the ABA-responsive element (ABRE), a conserved *cis*-element in the promoters of many ABA-responsive genes, including those of artemisinin biosynthesis. Considering that the SnRK2 family kinase can directly phosphorylate the ABRE-binding factors (especially the bZIP transcription factors), we searched for those genes encoding the putative SnRK2 proteins in *A*. *annua* by using the HMM search program against the sequenced transcriptomes of *A*. annua. Six candidate genes were identified based on their sequence similarity against the Arabidopsis and rice SnRK2 family (see Supplementary Fig. S1). Each of these genes was then isolated from the *A*. *annua* cDNAs. The deduced amino acid sequences of the six candidates showed homology with the SnRK2 family members from *A. thaliana*, but relatively lower homology with the SnRK2s from monocot species such as *Oryza sativa* (Supplementary Fig. S1). All the candidate SnRK2s of *A*. *annua* showed high similarity in the N-terminal regions, including the ATP-binding loop and the protein kinase-activating site (Supplementary Figs S1 and S2). A phylogenetic analysis revealed that the plant SnRK2 family had three groups (Fig. 1). Both Aannua05209S490740 and Aannua02702S329990 were included in Group I with AtSnRK2.1/4/5/9/10. The other four SnRK2 subfamily genes of *A*. *annua*, namely AaAPK1 and Aannua00085S022560/14917S778390/17791S810490, belonged to Group II, which included Arabidopsis SnRK2.2/3/6. Interestingly, ABA strongly activated the Group II members, such as Arabidopsis SnRK2.2, SnRK2.3, and SnRK2.6 (OST1), rather than the Group III members, which included SnRK2.7 and SnRK2.8 in Arabidopsis (Kulik *et al.* 2011).

### AaAPK1 physically interacts with AabZIP1

**Fig. 2.**
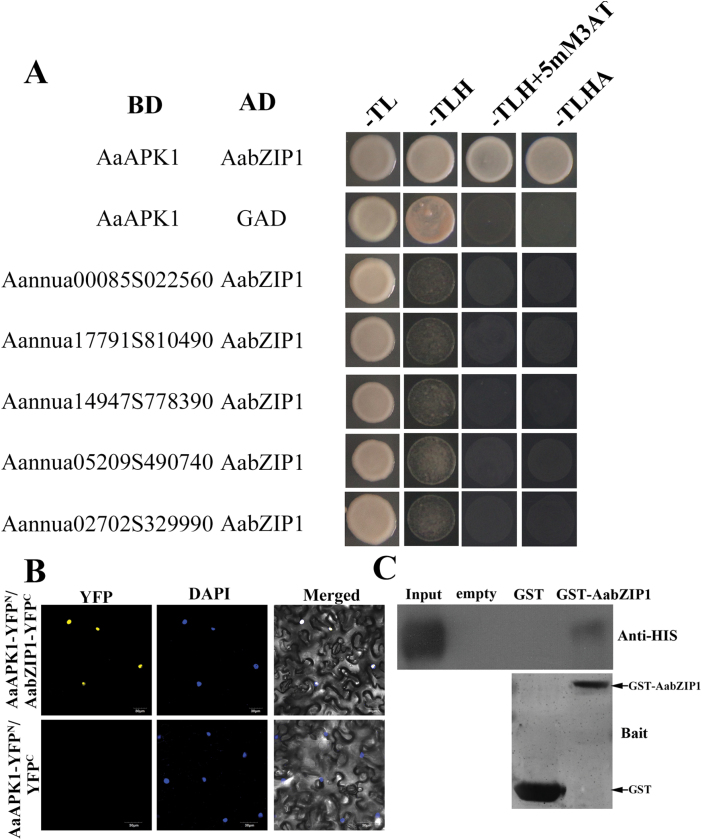
AaAPK1 interacts with AabZIP1. (A) Yeast two-hybrid assays showing the specific interaction between AaAPK1 and AabZIP1. BD, DNA-binding domain; AD, activating domain; –TL, synthetic medium without threonine and leucine; –TLH, synthetic medium without threonine, leucine, and histidine; –TLHA, synthetic medium without threonine, leucine, histidine, and adenosine. (B) Bimolecular fluorescence complementation (BiFC) assay to detect the interactions between AaAPK1 (fused with N-terminal fragment of yellow fluorescent protein, YFP) and AabZIP1 (fused with C-terminal fragment of YFP) in live plant cells. Construct pairs were co-infiltrated into leaves of *N*. *benthamiana*. The empty pEarleyagte202-YFP^C^ construct was used as a negative control. Nuclei were stained by DAPI. (C) Interaction between AaAPK1 and AabZIP1 in pull-down assays. HIS_6_-AaAPK1, GST, and GST-AabZIP1 were expressed in *E*. *coli* Rosetta (DE3) and purified. The proteins were pulled down by GST-AabZIP1 or GST and detected using anti-HIS_6_ antibody. GST-AabZIP1 or GST were stained by Coomassie Blue and shown as bait; AaAPK1-HIS^6^ was loaded as the input control. (This figure is available in colour at *JXB* online.)

To identify the interaction partners of AabZIP1, a yeast two-hybrid (Y2H) screening was employed. Each coding sequence of the six kinase candidates was cloned into the pGBKT7 vector fused with BD, while AabZIP1 was cloned into the pGADT7 vector fused with AD. The yeast cells harboring AaAPK1 and AabZIP1 grew well on the –TLHA selective medium (SD medium without Trp, His, Leu, and Ade). As the negative control, the yeast cells co-expressing the constructs empty pGADT7 and BD-AaAPK1 could not grow on the -TLHA selective medium. The yeast cells co-expressing one of the other five candidates and AabZIP1 were not able to grow on the -TLHA selective medium (Fig. 2A and Supplementary Fig. S3). This suggests that, among the six candidate kinases, only AaAPK1 physically interacted with AabZIP1. The interaction of AaAPK1 with AabZIP1 was further confirmed by the BiFC assay in tobacco cells (Fig. 2B). The N-terminus (amino acids 1–174) of YFP was fused with the C-terminus of AaAPK1, and the C-terminus (amino acids 175–239) of YFP was fused to the C-terminus of AabZIP1. Robust YFP fluorescence was detected in the nuclei of tobacco cells co-expressing AaAPK1-YFP^N^ and AabZIP1-YFP^C^ (Fig. 2B), but no such fluorescence was detected in cells co-expressing AaAPK1-YFP^N^ and YFP^C^. To further confirm the interaction between AaAPK1 and AabZIP1, a GST pull-down assay was done. The GST-AabZIP1 fusion protein was incubated with a GST affinity resin. As a result, GST-AabZIP1 could pull down AaAPK1-HIS^6^. As the negative control, GST could not pull down AaAPK1-HIS^6^ (Fig. 2C). Taken together, the results from the Y2H, BiFC, and pull-down assays demonstrate that AaAPK1 does physically interact with AabZIP.

### Expression analysis and subcellular localization of AaAPK1

**Fig. 3.**
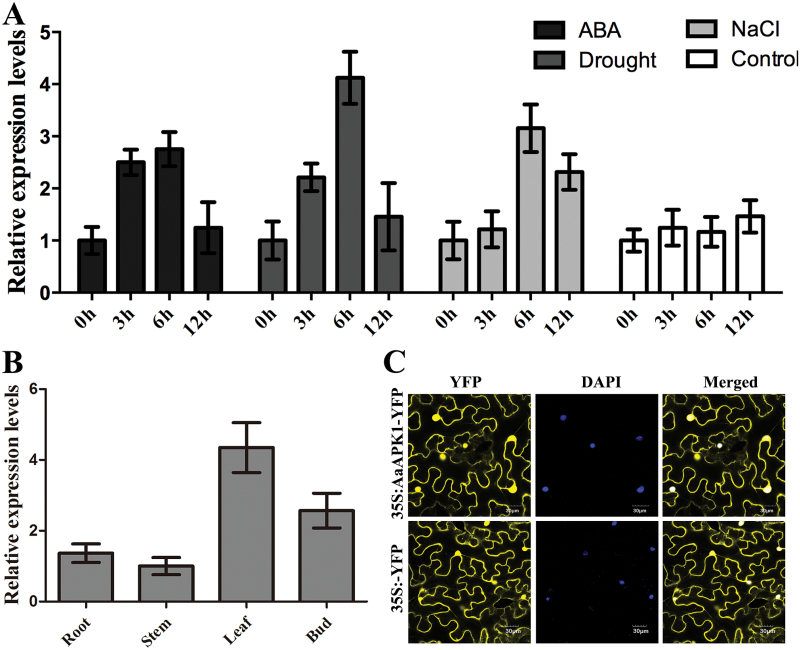
Expression analysis and subcellular localization of AaAPK1. (A) The expression levels of *AaAPK1* in under treatment with ABA, drought (air-drying), and NaCl. (B) The tissue profiles of *AaAPK1*. (C) Subcellular localization of AaAPK1. The coding sequence of AaAPK1 was in-frame fused with YFP, and under the control of the 35S promoter, then transferred into *A*. *tumefaciens* GV3101 and infiltrated into leaves of *N*. *benthamiana*. DAPI-stained nuclei were used as the control. (This figure is available in colour at *JXB* online.)

The expression levels of *AabZIP1* and the artemisinin biosynthesis genes become greatly elevated when the *A*. *annua* plants are treated with exogenous ABA, and when they are under drought or salt stress, and hence artemisinin biosynthesis is enhanced (Zhang *et al.*, 2014, 2015). ABA, drought, and salt stress also significantly up-regulated the *AaAPK1* expression level (Fig. 3A). Artemisinin is specifically synthesized in the glandular secretory trichomes of *A*. *annua* plants (Tan *et al.*, 2015). Young leaves and flower buds are rich in these trichomes and have higher expression levels of the artemisinin biosynthesis genes (Lu *et al.*, 2013). *AabZIP1* is also known to have higher expression levels in *A*. *annua* leaves and flower buds (Zhang *et al.*, 2015). In the present study, the tissue profiles showed that the expression levels of *AaAPK1* in the flower buds and leaves greatly exceeded those in the roots and stems (Fig. 3B). In addition, we examined the subcellular localization of AaAPK1 in tobacco cells using the transient expression of AaAPK1 fused with YFP. Robust fluorescence was observed in the nuclear and cytoplasmic compartments of cells expressing AaAPK1-YFP (Fig. 3C), suggesting that AaAPK1 is localized in both the nuclei and cytoplasm.

### AaAPK1 phosphorylates itself and AabZIP1

**Fig. 4.**
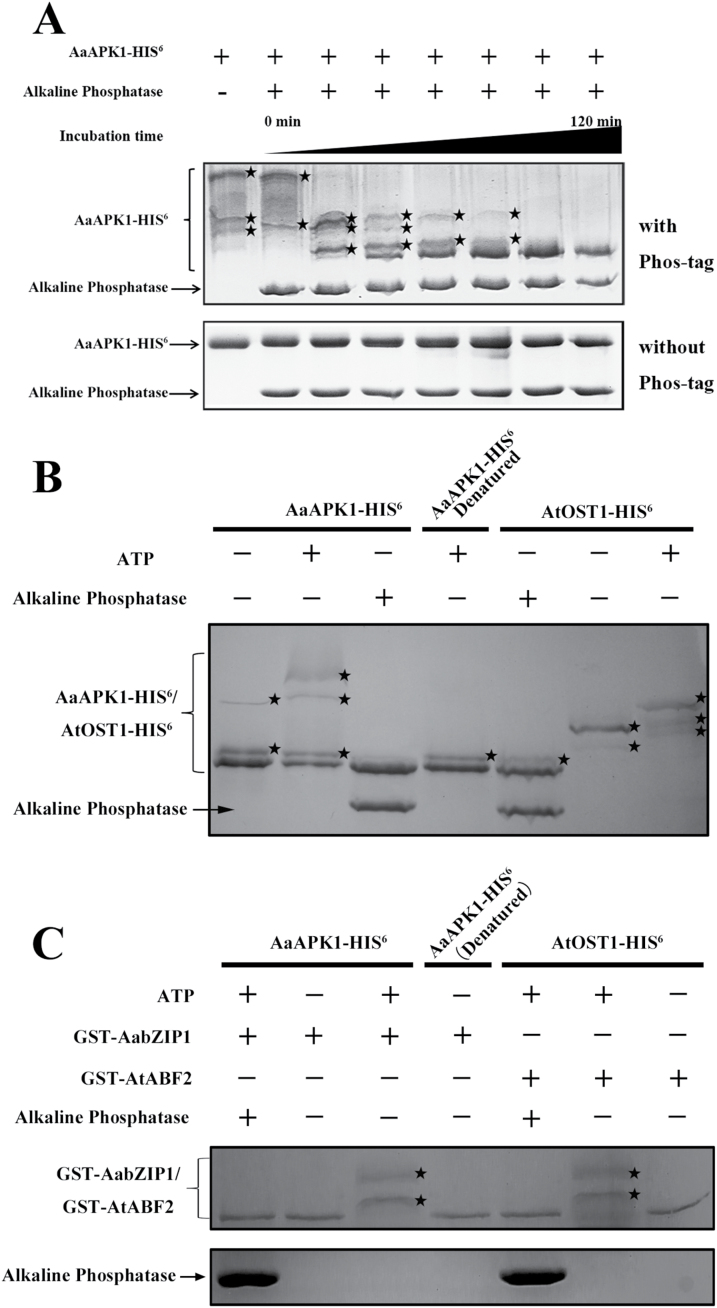
Phosphorylation of AabZIP1 by AaAPK1. HIS_6_-AaAPK1, GST, and GST-AabZIP1were expressed in *E*. *coli* Rosetta (DE3) and then purified. Phosphorylation of AaAPK1-HIS^6^ and GST-AabZIP1 were detected by a Phos-tag mobility shift assay. (A) *In vitro* kinase assay to detect autophosphorylation of AaAPK1. The AaAPK1-HIS^6^ was incubated in kinase assay buffer, either in the presence (+) or absence (–) of alkaline phosphatase and AaAPK1-HIS^6^, and the proteins were separated using 8% Phos-tag SDS-PAGE gel with Phos-tag (top panel) or without Phos-tag (bottom panel), then detected by Coomassie staining. Asterisks indicate the phosphorylated HIS_6_-AaAPK1. (B) *In vitro* kinase assay to detect autophosphorylation of AaAPK1. The AaAPK1-HIS^6^ or AtOST1-HIS^6^ (as indicated at the top) was incubated in kinase assay buffer, either in the presence (+) or absence (–) of alkaline phosphatase and ATP as indicated. (C) *In vitro* kinase assay to detect the phosphorylation of AabZIP1 by AaAPK1. The GST-AabZIP1 protein was incubated with AaAPK1 in kinase assay buffer, either in the presence (+) or absence (–) of alkaline phosphatase and ATP as indicated. GST-AtABF2 and AtOST1 were used as a positive control. After incubation, the proteins were separated using 8% Phos-tag SDS-PAGE gel with Phos-tag and detected by Coomassie staining. Asterisks indicate the phosphorylated GST-AabZIP1.

It has been demonstrated that auto-phosphorylated SnRK2s can phosphorylate several bZIP transcription factors (Kobayashi *et al.*, 2005; Ng *et al.*, 2011). To test whether AaAPK1 could phosphorylate itself, the Phos-tag mobility shift assay was employed, in which the binding of the phosphorylated-protein to the Phos-tag reagent in an SDS-PAGE gel slows down its movement (Bethke *et al.*, 2009). As shown in Fig. 4A, the AaAPK1-HIS^6^ protein displayed only one band in the gel without the Phos-tag, whereas at least four bands appeared with the Phos-tag. Moreover, after alkaline phosphatase (AP) treatment, the phosphorylation level of AaAPK1-HIS^6^ notably decreased with a longer incubation time (Fig. 4A). In the phosphorylation assays, Arabidopsis AtOST1 (namely AtSnRK2.6) was expressed in *E*. *coli* and then purified, which served as the positive control since its autophosphorylation has already been confirmed (Ng *et al.*, 2011). Our results for AtOST1 autophosphorylation, as seen in the Phos-tag assays, were consistent with a report on radio-labeling in gel kinase assays (Ng *et al.*, 2011). As shown in Fig. 4B, the phosphorylated AaAPK1-HIS^6^ increased significantly when ATP was present. Nevertheless, once AaAPK1-HIS^6^ was denatured through boiling, the phosphorylated band showed less AaAPK1-HIS^6^ in the kinase assay buffer without ATP (Fig. 4B); AaAPK1 exhibited similar results to AtOST1 under the same conditions. These results suggested that AaAPK1 is a protein kinase with the capacity of autophosphorylation.

The Phos-tag mobility shift assay was used to further investigate the phosphorylation of AabZIP1 by AaAPK1. For this, GST-AabZIP1 protein purified from the engineered *E. coli* was used as the substrate. The GST-AabZIP1 and AaAPK1-HIS^6^ recombinant proteins were incubated together in a kinase assay buffer. The movement of phosphorylated GST-AabZIP1 slowed down when AaAPK1-HIS^6^ and ATP were present. In addition, we used AtABF2 and AtOST1 from Arabidopsis as a positive control because the former is a proven phosphorylation substrate of latter (Wang *et al.*, 2013). The movement of GST-AtABF2 also slowed in the presence of AtOST1-HIS^6^ and ATP, consistent with previously reported work (Wang *et al.*, 2013). Either the alkaline phosphatase treatment or the absence of ATP inhibited the phosphorylation of GST-AabZIP1 by AaAPK1-HIS^6^, in addition to the phosphorylation of GST-AtABF2 by AtOST1-HIS^6^ (Fig. 4C). Together, these results indicated that AaAPK1 can phosphorylate AabZIP1 in an ATP-dependent manner.

### AaAPK1 enhances the transcriptional activity of AabZIP1

**Fig. 5.**
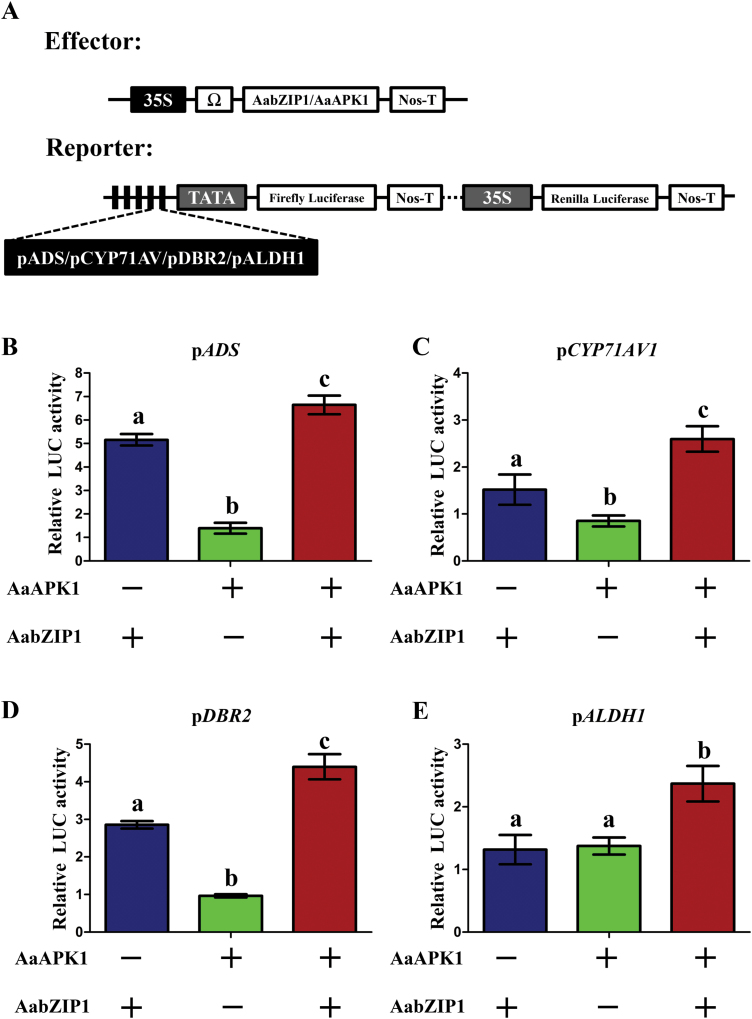
Dual-LUC assay of AaAPK1 and AabZIP1 transiently expressed in tobacco leaves. (A) Diagrams of the effector and reporter constructs used in the dual-LUC assay: both were transferred into *A*. *tumefaciens* GV3101 and co-infiltrated into leaves of *N*. *benthamiana*. The effects of AaAPK1, AabZIP1, and their combination on the activities of the promoters of the artemisinin biosynthesis genes *ADS* (B), *CYP71AV1* (C), DBR2 (D), and *ALDH1* (E) are shown as relative LUC activity. The values were determined by calculating the ratio of LUC activity to REN activity (LUC/REN) and then compared with the 35S::YFP control. Different letters indicate significant differences (*P*<0.05) as determined by *t*-tests. + indicates that the gene of interest was expressed in tobacco cells; – indicates that the gene of interest was not expressed. Data are means ±SD (*n*=3). (This figure is available in colour at *JXB* online.)

Given that AaAPK1 interacted with and then phosphorylated AabZIP1, we hypothesized that this phosphorylation by AaAPK1 may affect the functioning of AabZIP1 in the regulation of artemisinin biosynthesis. To test this hypothesis, we performed dual-LUC assays in tobacco leaves. The promoters of artemisinin biosynthesis genes (*ADS*, *CYP71AV1*, *DBR2*, and *ALDH1*) were each cloned into pGREEN-0800 and used as reporter constructs that expressed firefly luciferase (LUC) (Fig. 5A). The generated effector constructs expressed *AaAPK1* and *AabZIP1* under the control of 35S promoter. The pairs of effector and reporter constructs were transiently co-infiltrated into the leaves of *N*. *benthamiana*. As shown in Fig. 5, transfection of AabZIP1 alone resulted in the induction of *ProADS-LUC*, *ProCYP71AV1-LUC*, and *ProDBR2-LUC* (Fig. 5B–E), consistent with AabZIP1 being a positive regulator of the artemisinin biosynthesis genes (Zhang *et al.*, 2015). Transfection of AaAPK1 on its own could not induce the expression of *ProADS-LUC*, *ProCYP71AV1-LUC*, *ProDBR2-LUC*, and *ProALDH1-LUC*, but they were significantly induced when AaAPK1 was co-transfected with AabZIP1 (Fig. 5B–E). In particular, the transfection of AabZIP1 or AaAPK1 alone could not induce the expression of *ProALDH1,* whereas co-transfection of AabZIP1 with AaAPK1 significantly induced its expression. These results demonstrated that AaAPK1 enhanced the transcriptional activity of AabZIP1 for artemisinin biosynthesis genes, suggesting that AaAPK1 is a positive regulator of artemisinin biosynthesis.

### AaAPK1 phosphorylates serine^37^ of AabZIP1

**Fig. 6.**
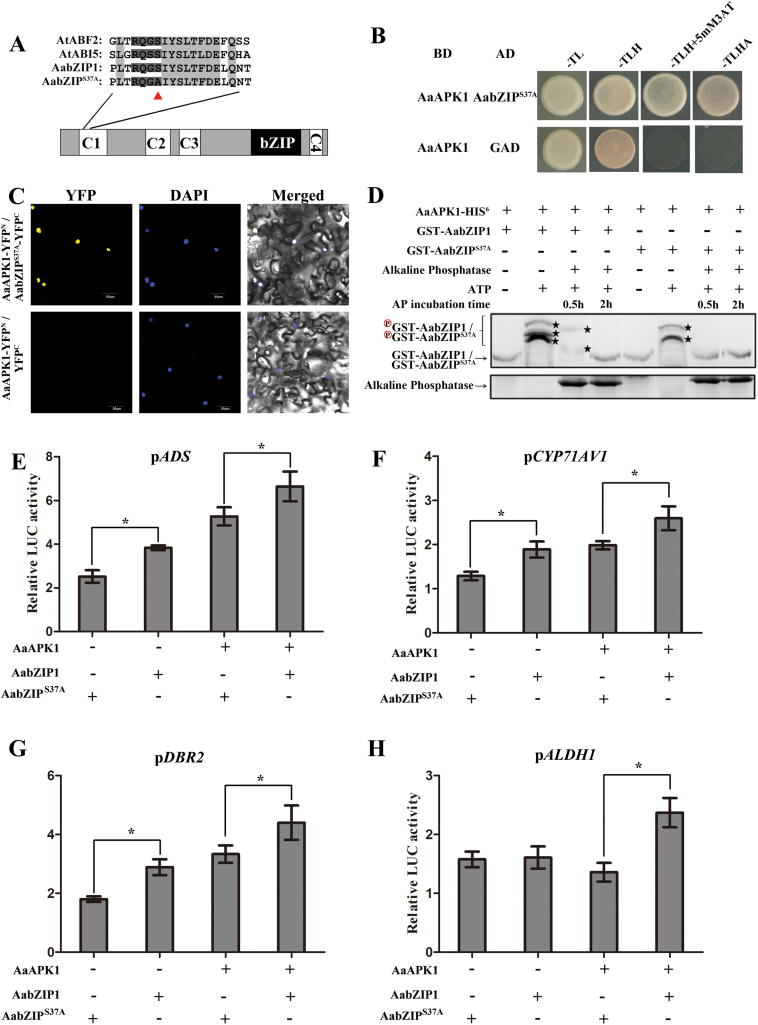
Substitution of Ser^37^ by Ala reduces the phosphorylation and the transactivation activity of AabZIP1. (A) Diagram of the AabZIP1 mutant. The polypeptides indicate the conserved protein kinase target sequences in the C1 domain of AREBs. The triangle indicates the location of the amino acid substitution. (B) A yeast two-hybrid assay was used to investigate the interaction of AabZIP1^S37A^ with AaAPK1. BD, DNA-binding domain; AD, activating domain; –TL: synthetic medium without threonine and leucine; –TLH: synthetic medium without threonine, leucine, and histidine; –TLHA: synthetic medium without threonine, leucine, histidine, and adenosine. (C) Bimolecular fluorescence complementation (BiFC) assay to detect the interactions of AaAPK1 (fused with N-terminal fragment of yellow fluorescent protein, YFP, in pEarleyagte201-YFP^N^) with AabZIP1^S37A^ (fused with C-terminal fragment of YFP in vector pEarleyagte202-YFP^C^). Construct pairs were co-infiltrated into leaves of *N*. *benthamiana*. The empty pEarleyagte202-YFP^C^ construct was used as a negative control. DAPI-stained nuclei were used as control. (D) *In vitro* kinase assay to compare the phosphorylation of AabZIP1 and AabZIP1^S37A^ by AaAPK1. The GST-AabZIP1or GST-AabZIP1^S37A^ protein was incubated with AaAPK1 in kinase assay buffer, either in the presence (+) or absence (–) of alkaline phosphatase and ATP. After incubation, the proteins were separated using 8% Phos-tag SDS-PAGE gel with Phos-tag and detected by Coomassie Blue staining. Asterisks indicate the phosphorylated GST-AabZIP1 or GST-AabZIP1^S37A^. (E–H) Transient transactivation analysis of AabZIP1^S37A^. Transient transactivation of the ADS, CYP71AV1, DBR2, and ALDH1 promoter-LUC fusion gene by AabZIP1 and the mutated AabZIP1^S37A^ by transiently expressing in tobacco leaves. The reporter is indicated at the top of each graph, and the presence (+) or absence (–) of the effectors is indicated at the bottom. The values were determined by calculating the ratio of LUC activity to REN activity (LUC/REN) and then compared with the 35S::YFP control. *, significant difference at *P*<0.05 as determined by *t*-tests. Data are means ±SD (*n*=3). (This figure is available in colour at *JXB* online.)

We know that SnRK2-type kinases phosphorylate Ser/Thr residues of R-X-X-S/T sites of AREB-type transcription factors (Furihata *et al.*, 2006). There were five R-X-X-S/T sites in AabZIP1 (see Supplementary Fig. S4). Only one of them (R-Q-G-S^37^), however, was localized in the C1 domain that is essential for the transcriptional activation of AabZIP1 (Zhang *et al.*, 2015). Moreover, this site was conserved in AabZIP1 and in other AREBs in Arabidopsis (Fig. 6A). To further investigate the phosphorylation of AabZIP1, we generated a point-mutant of AabZIP1 (AabZIP1^S37A^) by replacing the Ser^37^ in the R-X-X-S site of the C1 domain with an Ala^37^ (Fig. 6A). To exclude the possibility that the point-mutant in AabZIP1 interrupted the interaction between AabZIP1 and AaAPK1, Y2H and BiFC assays were performed. As shown in Fig. 6B, C, AabZIP1^S37A^ also physically interacted with AaAPK1. In addition, the phosphorylation level of AabZIP1 and AabZIP1^S37A^ were tested using a Phos-tag mobility shift assay. As shown in Fig. 6D, when ATP was absent, a single major band was observed in the lanes of both AabZIP1 and AabZIP1^S37A^. Interestingly, three clearly shifted bands of AabZIP1 were detected when compared with the two shifted bands that appeared in the AabZIP1^S37A^ lane. Moreover, AabZIP1^S37A^ gave only one band after 0.5 h of AP treatment, whereas the wild-type AabZIP1 retained two shifted bands. These results revealed that AaAPK1 partially phosphorylates AzbZIP1 at Ser^37^.

To investigate the effects of amino acid substitution at the phosphorylation sites of AabZIP1 on its transactivational activity, a transient dual-LUC assay was performed in tobacco leaves. As shown in Fig. 6E–H, when compared with those induced by the wild-type AabZIP1, the relative *pADS-LUC*, *pCYP71AV1-LUC*, and *pDBR2-LUC* activities as induced by AabZIP1^S37A^ were significantly decreased. Furthermore, co-transfecting AabZIP1^S37A^ with AaAPK1 resulted in significantly reduced relative activities of *pADS-LUC*, *pCYP71AV1-LUC*, *pDBR2-LUC*, and *pALDH1-LUC*, compared with the co-transfection of wild-type AabZIP1 and AaAPK1. Together, these results suggested that the Ser^37^ phosphorylation of AabZIP1 by AaAPK1 is important for activating the AabZIP1-mediated transcriptional regulation.

### Effects of AaAPK1 on artemisinin biosynthesis

**Fig. 7.**
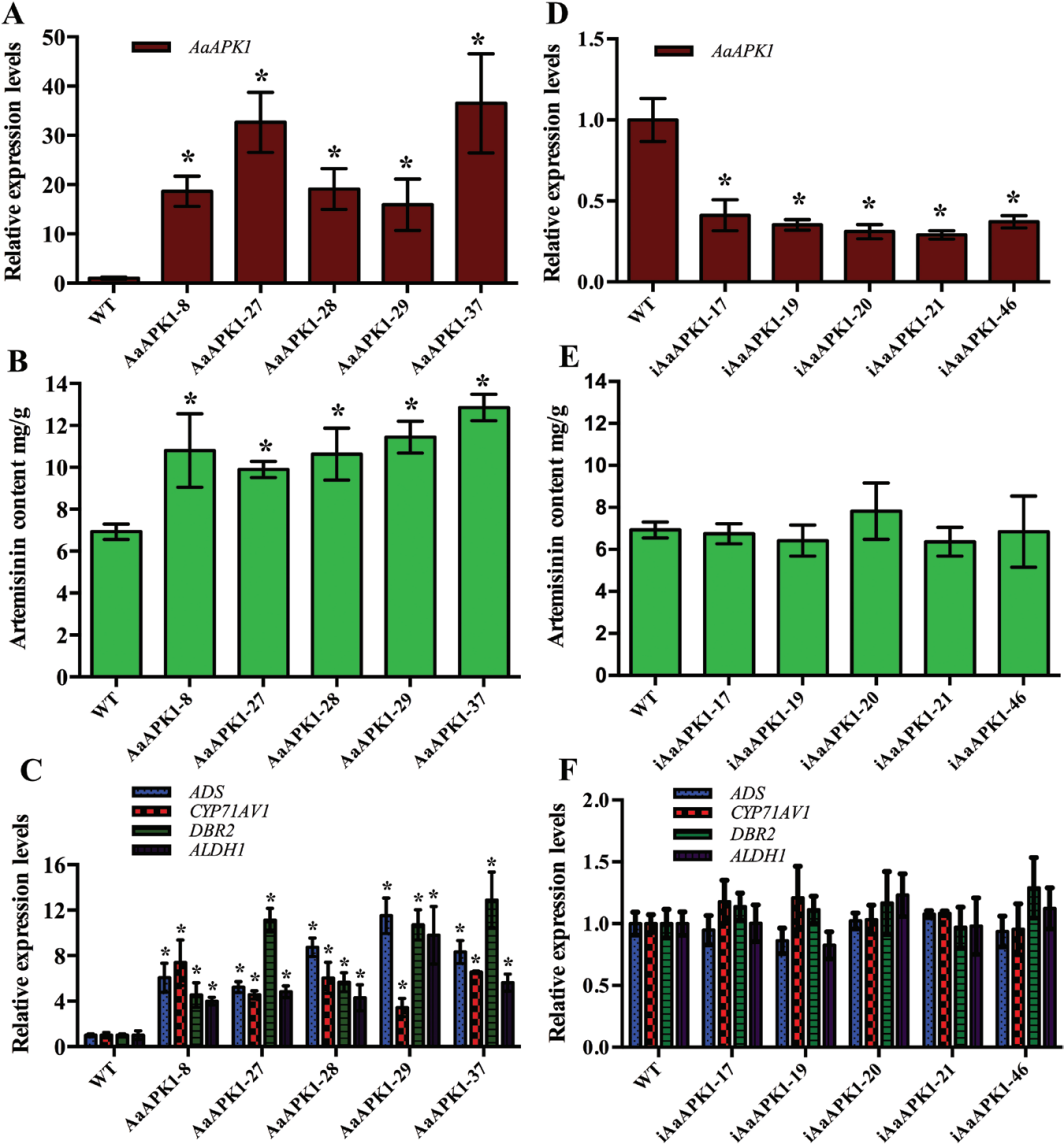
Analysis of gene expression and artemisinin content in *A*. *annua*. (A) Relative expression level of *AaAPK1* in *AaAPK1*-overexpressing and wild-type (WT) plants. (B) Artemisinin content in *AaAPK1*-overexpressing and WT plants. (C) Relative expression levels of artemisinin biosynthesis genes in *AaAPK1*-overexpressing and WT plants. (D) Relative expression level of *AaAPK1* in *AaAPK1*-RNAi and WT plants. (E) Artemisinin content in *AaAPK1*-RNAi and WT plants. (F) Relative expression levels of artemisinin biosynthesis genes in *AaAPK1*-RNAi and WT plants. *, significant difference at *P*<0.05 as determined by *t*-tests. Data are means ±SD (*n*=3). (This figure is available in colour at *JXB* online.)

Transgenic plants of *A*. *annua* in which *AaAPK1* was overexpressed were established to investigate the effects of its expression on artemisinin biosynthesis. The fragments of 35S::AaAPK1 were amplified from both the positive control (PHB-AaAPK1 plasmid) and the transgenic plants with AaAPK1 overexpression, but they were not detected in the wild-type plants of *A*. *annua* (see Supplementary Fig. S5). The *AaAPK1*-overexpressing plants had expression levels that were 15.9- to 36.5-fold those of the wild-type plants (Fig. 7A). A metabolite analysis showed that these *AaAPK1*-overexpressing plants produced artemisinin at higher levels than did the wild-type plants (Fig. 7B), consistent with the higher expression levels of the artemisinin biosynthesis genes, *ADS*, *CYP71AV1*, *DBR2*, and *ALDH1* (Fig. 7C). The *AaAPK1*-overexpressing plants produced artemisinin in the range of 9.89 to 12.85 mg g^–1^ dry weight (DW), whereas the 15 independent wild-type plants produced it at an average level of 6.93 mg g^–1^ DW about 53.9–70.1% that of the transgenic plants.

Using RNAi technology, we also developed transgenic plants of *A*. *annua* in which AaAPK1 was suppressed. The authentic plants of AaAPK1-RNAi *A*. *annua* were confirmed by PCR (see Supplementary Fig. S6) and qPCR. The *AaAPK1*-RNAi plants were confirmed as having much lower expression levels of *AaAPK1* (Fig. 7D) without significantly down-regulating the production of artemisinin. Both this production and the expression levels of artemisinin biosynthesis genes were similar between the *AaAPK1*-RNAi and wild-type plants (Fig. 7E, F), probably due to the redundant SnRK2 family members in *A*. *annua* that might have rescued the RNA interference effect of *AaAPK1*. The results of the transgenic overexpression of *AaAPK1* indicated that it is a valuable gene for breeding high-yielding artemisinin plant varieties of *A*. *annua*.

## Discussion

SnRK2-type kinases play an important role in the ABA and osmotic-stress signaling pathways. The autophosphorylation of AaSnRK2s, and the ensuing phosphorylation of downstream proteins to activate ABA-responsive gene expression, is a key process in ABA-signaling transduction (Kobayashi *et al.*, 2005, Furihata *et al.*, 2006, Sato *et al.*, 2009). Although the molecular mechanism of the involvement of SnRK2 kinases in osmotic stress signaling is not yet clear, phenotype analysis of an Arabidopsis decuple-mutant has shown that they are important regulators in the signaling pathway (Fujii *et al.*, 2011). Thus, the SnRK2 family kinases are regarded as an important target when breeding drought-resistant plants. A series of SnRK2 homologs have been characterized from Arabidopsis, rice, maize, and other plants (Kulik *et al.*, 2011). Although we have made rapid progress in understanding the function of SnRK2 kinases in ABA and osmotic-stress signaling, little is known about the mechanisms by which they act in regulating secondary metabolite biosynthesis, especially at the post-transcription level. In the case of artemisinin, its production is clearly promoted when *A*. *annua* plants are treated with ABA or NaCl (Jing *et al.*, 2009, Wang *et al.*, 2016). Previously, we characterized an ABA-responsive transcription factor, AabZIP1, that positively regulates artemisinin biosynthesis by directly binding and activating the promoters of the artemisinin biosynthesis genes (Zhang *et al.*, 2015). In the present study, we report on an SnRK2-type (AaAPK1) and its involvement in regulating artemisinin biosynthesis through phosphorylation of AabZIP1.

A high redundancy of SnRK2s occurs in plants; for example, there are at least 10 members in Arabidopsis (SnRK2.1–SnRk2.10) and 10 in *Oryza sativa* (SAPK1–SPAK10) (Fujii *et al.*, 2011). We found six SnRK2 members in *A. annua*, according to its transcriptome data. However, only AaAPK1 interacted with AabZIP1 among the six candidates obtained by our Y2H screening (Fig. 2A). This interaction was further confirmed by BiFC and pull-down assays. These results therefore suggest that AaAPK1 may regulate artemisinin biosynthesis through its interaction with AabZIP1. *In silico* analysis of amino acid sequences showed that AaAPK1 possessed all the basic features of the SnRK2 kinases, namely the ATP binding loop, the activation loop, the conserved PP2C interface residues, and the ABA box (Ng *et al.*, 2011). Phylogenetic analysis of AaAPK1 with its closely related SnRK2s suggested that it belonged to Group II, which was composed of ABA-induced SnRK2 family members (Kulik *et al.*, 2011). Interestingly, the expression of AaAPK1 was up-regulated by drought or NaCl treatment, and it exhibited similar expression patterns to many of the known key genes and regulators of the artemisinin biosynthetic pathway. Notably, *AaAPK1* exhibited responses to ABA, not unlike the AaPP2C1, AabZIP1, and the artemisinin biosynthesis genes did (Zhang *et al.*, 2014, 2015). The ABA-induced *AaAPK1* expression confirmed the phylogenetic result of AaAPK1 belonging to the ABA-responsive Group II of the SnRK2 subfamily.

Based on the results from the Y2H, BiFC, and GST pull-down assays, the interaction between AaAPK1 and AabZIP1 was confirmed. We suspected that AabZIP1 is the substrate of AaAPK1, because the bZIP transcription factors, such as AREB1, ABI5, and AREB3, are all substrates of SnRK2 kinase (Furihata *et al.*, 2006; Fujii *et al.*, 2007; Feng *et al.*, 2014). The phosphorylated bZIP proteins could well activate the downstream target genes (Nakashima *et al.*, 2009; Wang *et al.*, 2013). Disruption of the three protein kinases, namely SnRK2.2, 2.3, and 2.6, could down-regulate the ABA-inducible gene expression, including that of the ABI5, AREB1, and AREB3 target genes (Nakashima *et al.*, 2009). Moreover, ABI5 could promote transcription of *FLOWERING LOCUS C* (*FLC*) by binding ABRE/G-box in the *FLC* promoter, an activity that is increased by overexpressing OST1 (Wang *et al.*, 2013). In our present study, the function of AabZIP1 as the phosphorylation substrate of AaAPK1 was confirmed by a Phos-tag mobility shift assay (Fig. 4C), suggesting that AabZIP1 was regulated via phosphorylation at the post-translational level. The Ser/Thr residue of the R-X-X-S/T sites is one of the most important phosphorylation sites of the AREB-type activator in Arabidopsis (Furihata *et al.*, 2006): we found that the Ser^37^ of AabZIP1 was an important amino acid, and one that was phosphorylated by AaAPK1 (Fig. 6). The Ser^37^ residue of the R-Q-G-S^37^ site was in the C1 domain of AabZIP1, and this particular domain is essential for AabZIP1 transactivation of the artemisinin biosynthesis genes (Zhang *et al.*, 2015). Overexpression of the AabZIP1 deletion mutant (i.e. AabZIP1∆C1) in the dual-LUC analysis, previously performed by Zhang *et al.* (2015), had a negligible influence on the reporter activity, irrespective of the ABA treatment applied.

Since AaAPK1 had a similar expression pattern to AabZIP1 and the phosphorylated AabZIP1, this led us to study the effect of AaAPK1 on the AabZIP1 transcriptional activity for the artemisinin biosynthesis genes. The dual-LUC analysis we performed here in tobacco leaves showed that on its own the transient overexpression of *AaAPK1* did not increase the transcription of artemisinin biosynthesis genes. A plausible explanation for this result is that AaAPK1, being a kinase, could not directly bind with the DNA. As previously reported by Zhang *et al.* (2015), the transcription of *ADS*, *CYP71AV1*, and *DBR2* was significantly elevated when AabZIP1 was transiently overexpressed. However, the transcription of the artemisinin biosynthesis genes was further elevated by transiently overexpressing AaAPK1 and AabZIP1 together, when compared with the transient overexpression of AabZIP1 alone (Fig. 5). Moreover, the Ser^37^ of the AabZIP1 mutant (AabZIP1^S37A^) had a reduced transcriptional activity when compared with AabZIP1 (Fig. 6E–H). Based on these findings, we deduced that AabZIP1 enhanced its transcriptional activity on the artemisinin biosynthesis genes when it was phosphorylated, and that AaAPK1 participated in regulating artemisinin biosynthesis by phosphorylating AabZIP1.

It should be possible to increase artemisinin production by engineering its biosynthetic pathway and its regulating transcription factors. Overexpression of the key enzymes involved in the 2C-methyl-D-erythritol 4-phosphate (MEP) pathway, the mevalonate pathway, and the artemisinin-specific biosynthetic pathways (and their combinations) was found to promote artemisinin biosynthesis, leading to the successful establishment of *A*. *annua* plants with a high yield of artemisinin (Tang *et al.*, 2014). In addition to the key enzymes and metabolite-regulating transcription factors, the metabolite-regulating kinases could also be used to promote biosynthesis through the overexpression method. To date, however, very few kinases have been identified and used to enhance secondary metabolite biosynthesis, because our knowledge of their participation in regulation is limited. The MAP-kinase kinase 1 of *C*. *roseus* (CrMAPKK1) is a typical example. In root cultures of *C*. *roseus*, overexpression of *CrMAPKK1* elevates the expression levels of the terpenoid indole alkaloid (TIA) biosynthesis genes and increases the accumulation of TIAs such as tabersonine, ajmalicine, and catharanthine (Paul *et al.*, 2017). AaAPK1 was the first kinase identified to positively regulate artemisinin biosynthesis. Overexpression of *AaAPK1* significantly up-regulated the expression levels of biosynthesis genes and increased the production of artemisinin in the transgenic plants of *A*. *annua*. However, in the present study, the artemisinin contents showed little difference between the AaAPK1-suppressed transgenic lines and the wild-type plants. The expression levels of artemisinin biosynthesis genes, as well as the other five SnRK2 members in *A*. *annua*, were not significantly different between AaAPK1-RNAi and wild-type plants (Fig. 7F and Supplementary Fig. S7). This could be explained by the high redundancy of the SnRK2 family in plants. There are 10 members of the SnRK2 family in Arabidopsis (SnRK2.1–2.10) and *Oryza sativa* (SAPK1–10). When nine SnRK2s were knocked-out in Arabidopsis, the mutants grew normally in soil while the decuple-mutants showed a clear growth defect (Fujii *et al.*, 2011). Furthermore, the constitutive expression of AtSnRK2.6 greatly raised the sucrose and total soluble sugar levels in Arabidopsis leaves; however, the soluble sucrose was similar between the *snrk2*.*6* mutant and its null sibling (Zheng *et al.*, 2010). Thus, the high redundancy of SnRK2s appears to complicate the dissection of SnRk2-member functionality through reverse genetics. Considering that *A*. *annua* belongs to the Asteraceae plant family, its genome (approx. 1.8 G, unpublished data) is perhaps much more complex than is the smaller-sized Arabidopsis genome. Conceivably, then, *A*. *annua* may harbor more than six SnRK2 genes. Exploring the genome of *A*. *annua* in greater detail will prove invaluable for investigating the function of individual members in SnRK2 and other important gene families.

In conclusion, an ABA-responsive SnRK2-type kinase, AaAPK1, was identified and found to be involved in positively regulating artemisinin biosynthesis through phosphorylation of AabZIP1. This functional characterization of AaAPK1 not only facilitates a deeper understanding of the regulatory mechanism underpinning artemisinin biosynthesis at the post-translational level, but it also provides a novel and timely candidate gene for engineering the production of artemisinin.

## Supplementary data

Supplementary data are available at *JXB* online.

Table S1. Primers used in this study.

Fig. S1. Alignment of six SnRK2 kinase family candidates with SnRK2 family members from Arabidopsis and rice.

Fig. S2. Alignment AaAPK1 with SnRK2.2/2.3/2.6 from Arabidopsis.

Fig. S3. Self-activation test of six kinase candidates in a Y2H assay.

Fig. S4. Diagram of the putative SnRk2-type kinase target in AabZIP1.

Fig. S5. PCR detection of positive AaAPK1-overexpression *A. annua* and artemisinin content analysis of 10 independent transgenic lines.

Fig. S6. PCR detection of positive AaAPK1-RNAi *A. annua* and artemisinin content analysis of 10 independent AaAPK1-RNAi lines.

Fig. S7. Relative expression levels of *Aannua00085S022560*, *Aannua17791S810490*, *Aannua14947S778390*, *Aannua05209 S490740*, and *Aannua02702S329990* in AaAPK1-RNAi trans genic lines and wild-type *A. annua*.

Supplementary Figures S1-S7
