# Supplementary material for: ARTEMISININ BIOSYNTHESIS PROMOTING KINASE 1 positively regulates artemisinin biosynthesis through phosphorylating AabZIP1
Source: J Exp Bot. 2017 Dec 28;69(5):1109–23. doi: 10.1093/jxb/erx444 (PMC6019033; doi:10.1093/jxb/erx444)
Supplement: Supplementary Figures S1-S7 [file erx444_suppl_supplementary_figures_s1-s7.pdf]

Supplementary Table S1 Primers used in this study

Primers	Sequence 5'-3'	Purpose
AaAPK1-qF1	AGGAGCCCGATCAACCTATG	qRT-PCR
AaAPK1-qR1	CAGAGTCAAAATCGTCCAGATC	
AaADS-qF	AATGGGCAAATGAGGGACAC	qRT-PCR
AaADS-qR	TTTCAAGGCTCGATGAACTATG	
AaCYP71AV1-qF	CACCCTCCACTACCCTTG	qRT-PCR
AaCYP71AV1-qR	GACACATCCTTCTCCCAGC	
AaDBR2-qF	CTTGGGTTACAAGCTGTGGCTCAAG	qRT-PCR
AaDBR2-qR	ATATAATCAAAACTAGAGGAGTGACC	
AaALDH1-qF	CAGTTTCTGACCCAAATCCAGGTTGA	qRT-PCR
AaALDH1-qR	TCGGAGTAGTTGGTCACAT	
Aannua00085S022560-F-cacc	caccATGGATCGATTACCGGGGAT	gateway pEntry cloning
Aannua00085S022560-R	CATAGCATAAACGATTTCAC	
Aannua17791S810490-F-cacc	caccATGGACAACAGATCTATGCT	gateway pEntry cloning
Aannua17791S810490-R	CATAGCGTATACAATCTCCC	
Aannua14947S778390-F-cacc	caccATGGACAACAGATCTATGCTGAC	gateway pEntry cloning
Aannua14947S778390-R	TCGACACTCTGAGTCGGCTG	
Aannua05209S490740-F-cacc	caccATGGACAAGTATGAGGTGGTAAAG	gateway pEntry cloning
Aannua05209S490740-R	ATCATCTTCTTCTGCTCCCC	
Aannua02702S329990-F-cacc	caccATGGAGAAGTATGAGTTAGT	gateway pEntry cloning
Aannua02702S329990-R	TGGGATGGGCGGGATTTCTC	
AaAPK1-F-cacc	caccATGGATCGGAATATGAGTTC	gateway pEntry cloning
AaAPK1-R	GATGGCGTATATGACCTCAC	
AaAPK1-RI-cacc	caccGTGGTTTCTGAAGAACTTAC	gateway pEntry cloning
AaAPK1-RI-R	GGGTCCAGTCTTTTTTCCAA	
AaAPK1-F-EcoRI	GCGAATTCATGGATCGGAATATGAGTTC	Rrotein recombinant
AaAPK1-R-XhoI	GCCTCGAGGATGGCGTATATGACCTCAC	
AabZIP1-F-EcoRI	GCGAATTCATGAACTACAAGAATTTTGG	Rrotein recombinant
AabZIP1-R-XhoI	GCCTCGAGCCATGGACCGGAAAGTGTCT	
AaABI1-F-EcoRI	CGGAATTCATGGAAGATATCCCTCCTTCGG	Yeast two hybrid
AaABI1-R-SacI	CGGAGCTCGTCAAGATTTAGTTTTAAACC	
AaSnRK2.2-F-BamHI	GCGGATCCATGGATCGATTACCGGGGAT	Yeast two hybrid
AaSnRK2.2-R-SalI	GCGTCGACCATAGCATAAACGATTTCAC	
AaSnRK2.3-F-SalI	GCGTCGACATGGACAACAGATCTATGCT	Yeast two hybrid
AaSnRK2.3-R-PstI	GCCTGCAGCATAGCGTATACAATCTCCC	
AaSnRK2.4-F-SalI	GCGTCGACATGGACAACAGATCTATGCTGA	Yeast two hybrid
AaSnRK2.4-R-PstI	GCCTGCAGTCGACACTCTGAGTCGGCTG	
AaSnRK2.5-F-SalI	GCGTCGACATGGACAAGTATGAGGTGGTAA	Yeast two hybrid
AaSnRK2.5-R-PstI	GCCTGCAGATCATCTTCTTCTGCTCCCC	
AaSnRK2.6-F-SalI	GCGTCGACATGGAGAAGTATGAGTTAGT	Yeast two hybrid
AaSnRK2.6-R-PstI	GCCTGCAGTGGGATGGGCGGGATTTCTC	
AaAPK1-F-PstI	GCCTGCAGATGGATCGGAATATGAGTTC	Overexpression
AaAPK1-R-BclI	GCTGATCAGATGGCGTATATGACCTCAC	

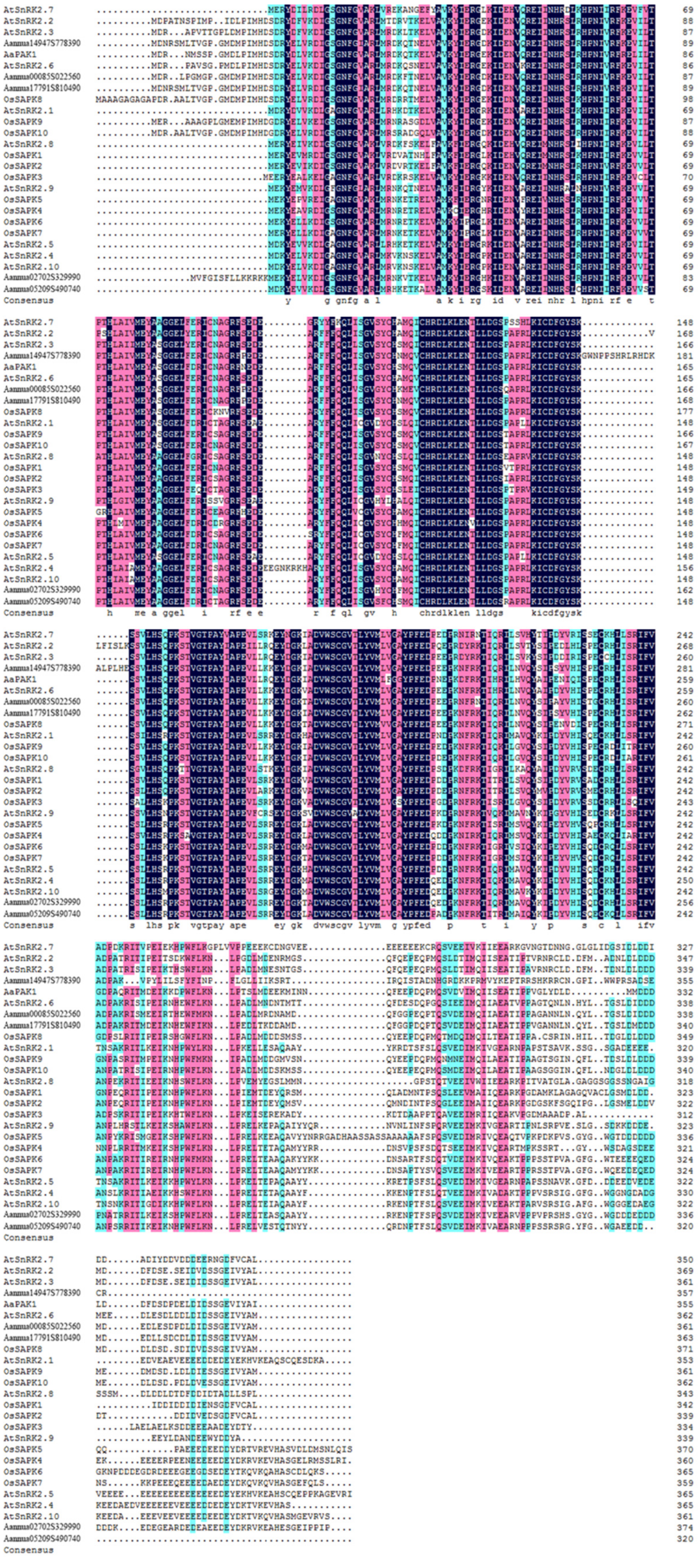

Fig. S1 Alignment of six SnRK2s kinase family candidates with SnRK2S family members from *Arabidopsis* and Rice.

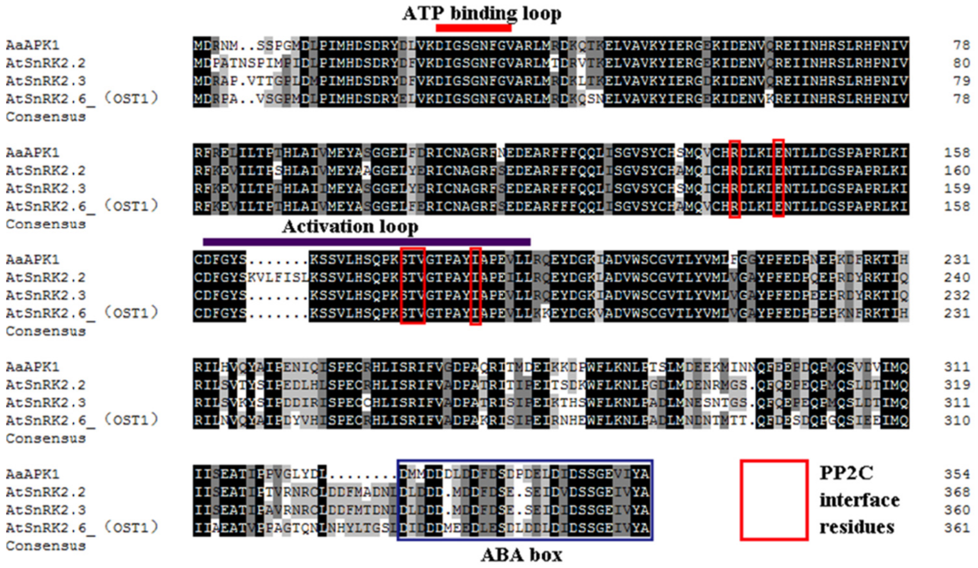

Fig. S2 Alignment AaAPK1 with SnRK2.2/2.3/2.6 from *Arabidopsis*. The under the red line represented the conserved ATP binding loop in SnRK2s family; the sequences under the purple line represented the activation loop in SnRK2s family; the sequences in the red box represented the amino acid residues for PP2C interface; the sequences in the blue box represented the domain for ABA response.

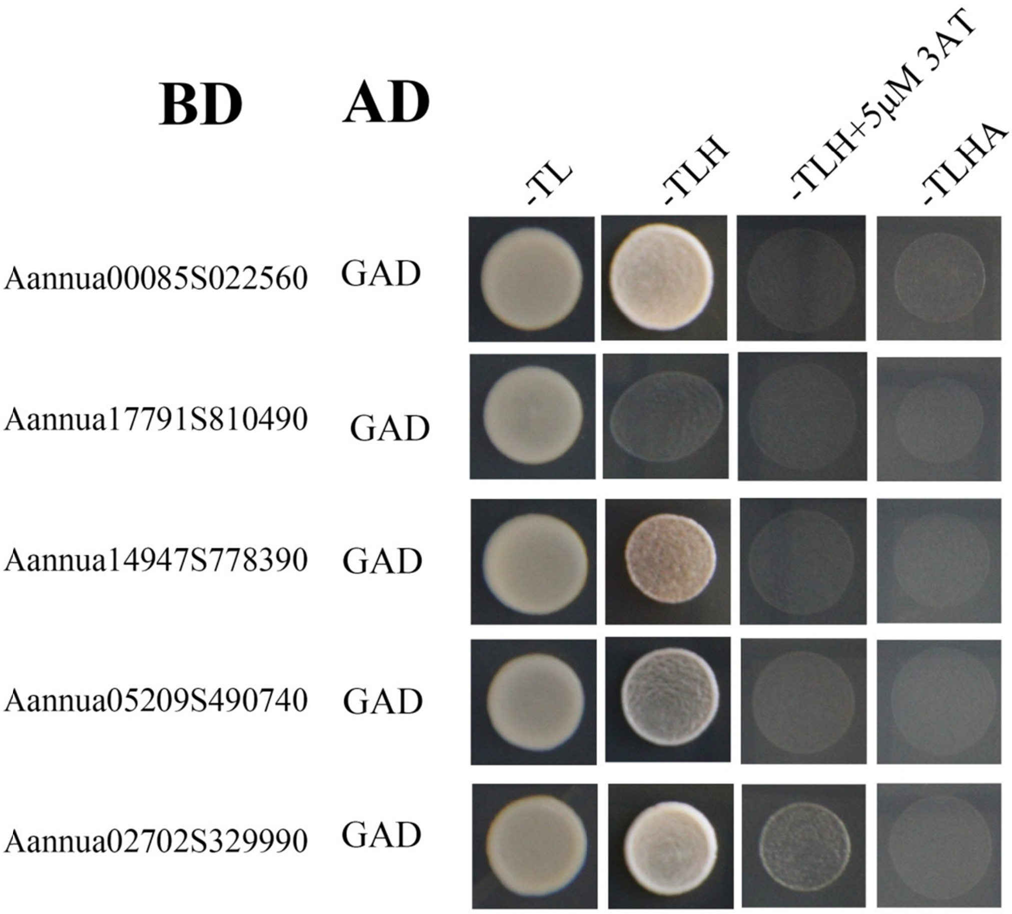

Fig. S3 The self-activation test of six kinase candidates in Y2H assay. The BD represented the candidate genes cloned into pGBKT7 vector, while the GAD represented the empty pGADT7 vector. -TL: synthetic medium dropout threonine and histidine; -TLH: synthetic medium drop out threonine, leucine and histidine; -TLHA: synthetic medium dropout threonine, leucine, histidine and adenosine.

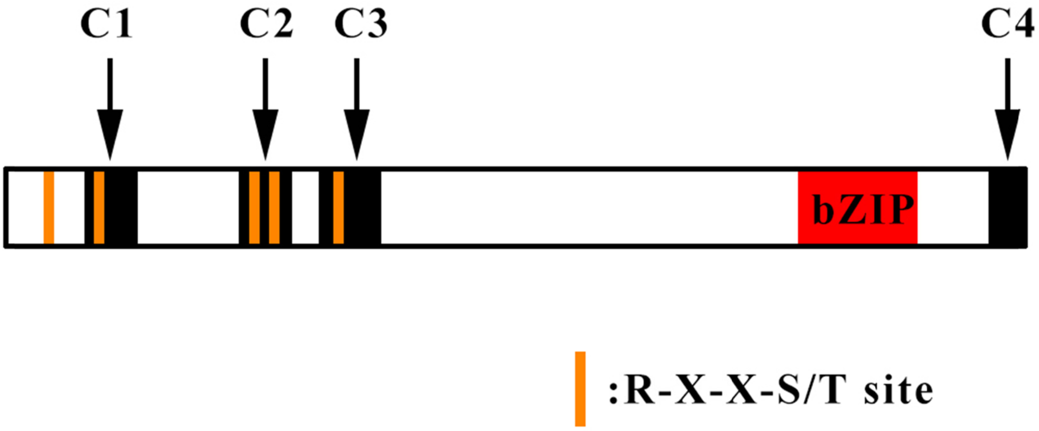

Fig. S4 The Schemes of putative SnRk2 type kinase target in AabZIP1. The yellow histogram represent the R-X-X-S/T site in AabZIP1.

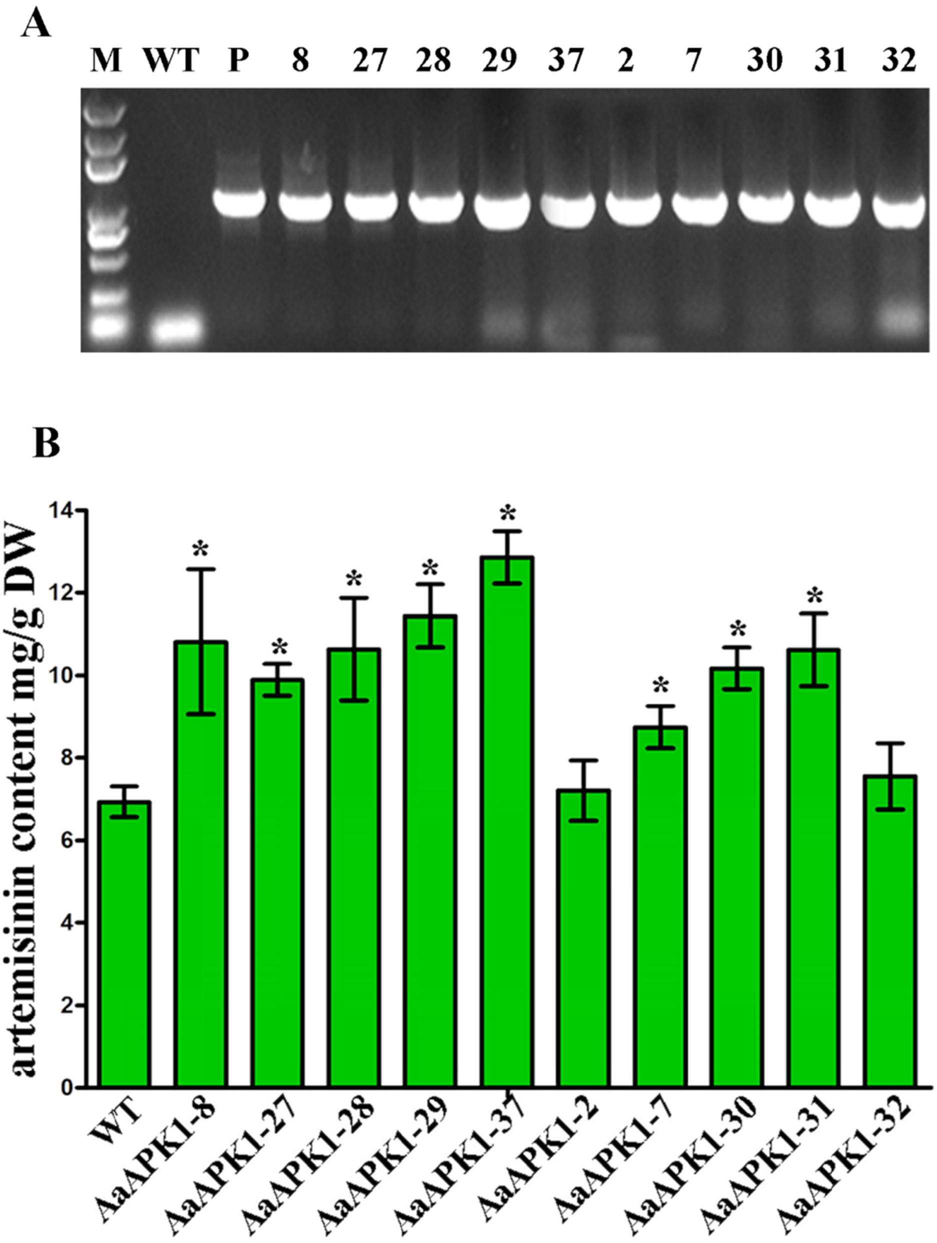

Fig. S5 The PCR detection of positive AaAPK1 overexpression *Artemisia* and artemisinin contents analysis of 10 independent transgenic *Artemisia*. A, The PCR detection of positive transgenic *Artemisia* using genomic DNA. M: marker; WT: wild type *Artemisia*, used as negative control; P: PHB-AaAPK1 plasmid, used as positive control. B: the artemisinin contents in *AaAPK1*-overexpressing and wild type Artemisia. WT: wild-type *A. annua* plants. *: significant difference at the level of $p<0.05$ given by t-test. Error bars represent ±SD (n=3).

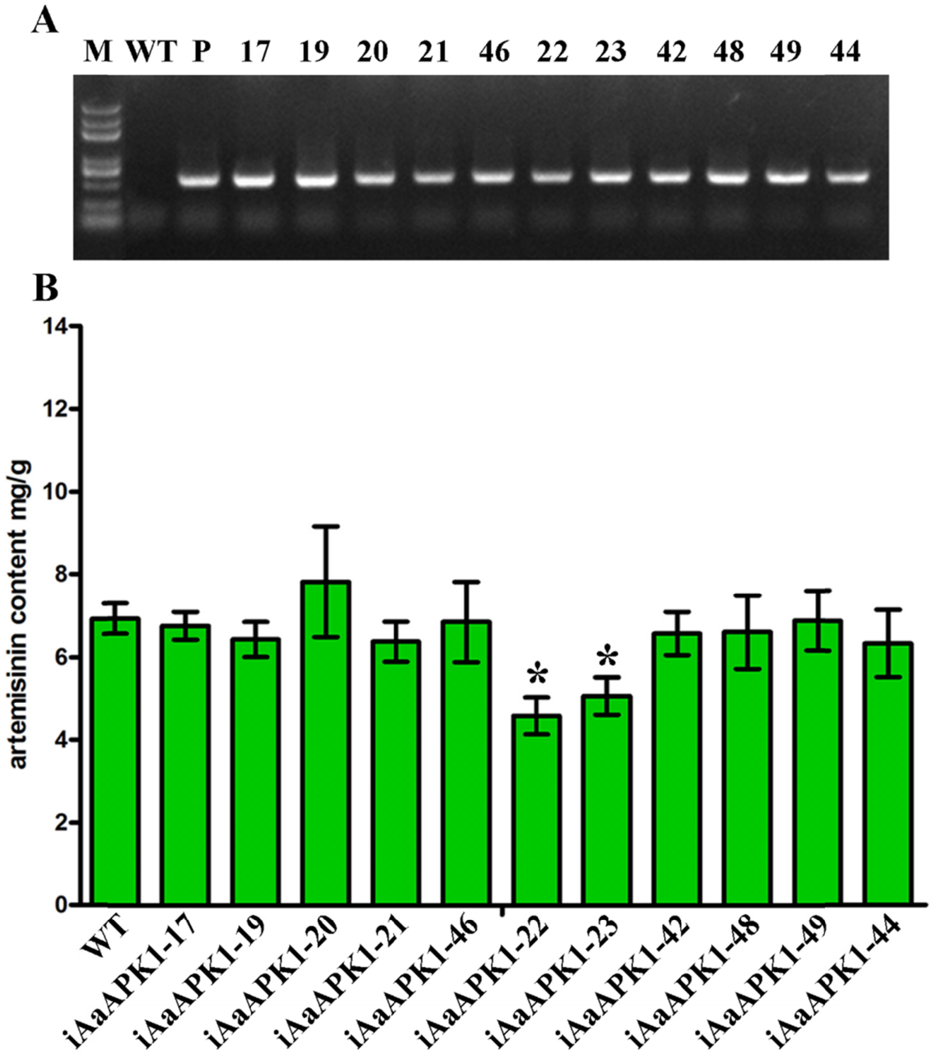

Fig. S6 The PCR detection of positive AaAPK1-RNAi *Artemisia* and artemisinin contents analysis of 10 independent AaAPK1-RNAi *Artemisia*. A, The PCR detection of positive transgenic *Artemisia* using genomic DNA. M: marker; WT: wild type *Artemisia*, used as negative control; P: pHELLSGATE-iAaAPK1 plasmid, used as positive control. B: the artemisinin contents in AaAPK1-RNAi and wild type *Artemisia*. WT: wild-type *A. annua* plants. *: significant difference at the level of $p<0.05$ given by t-test. Error bars represent ±SD (n=3).

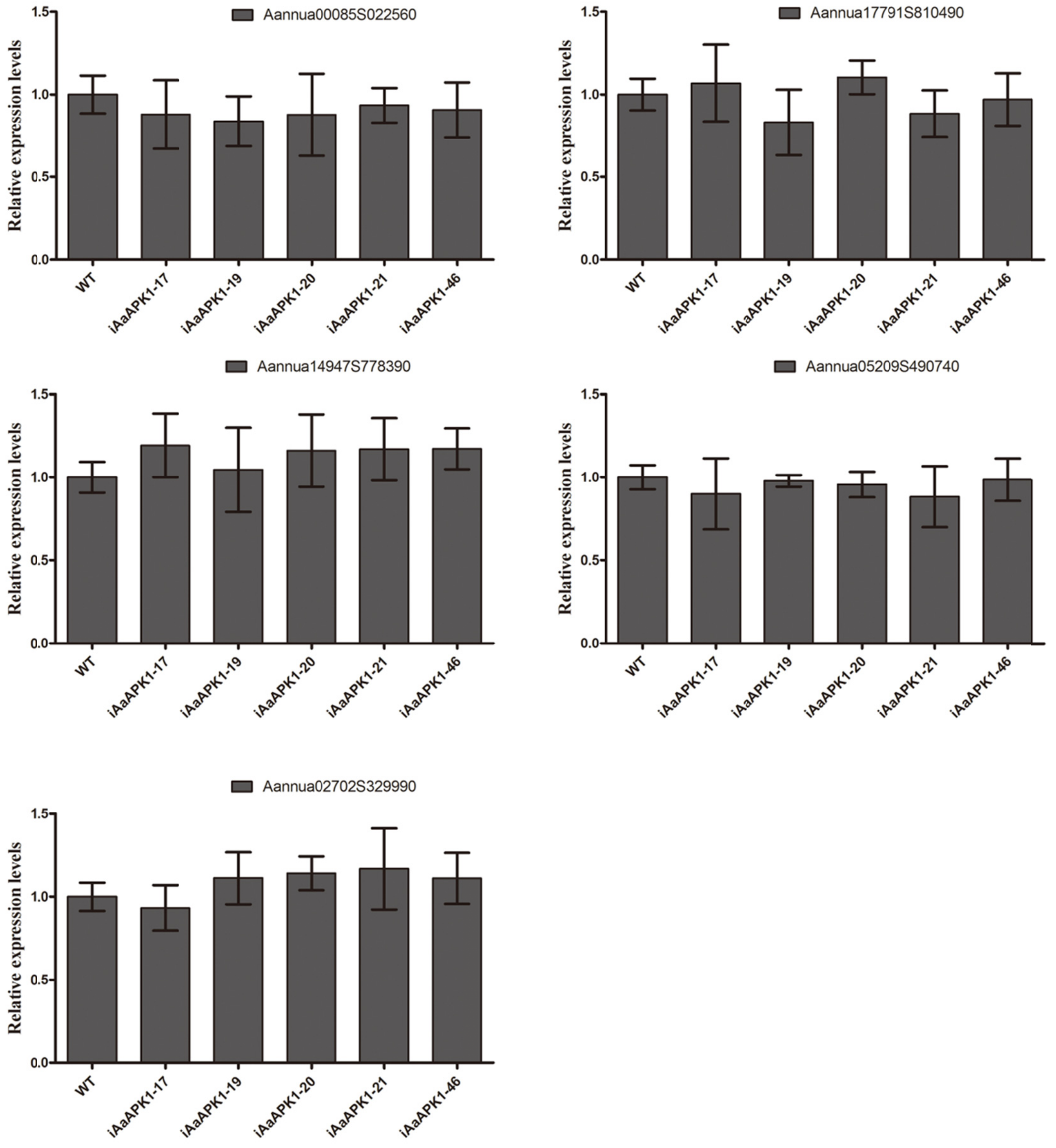

Fig. S7 The relative expression levels of *Aannua00085S022560*, *Aannua17791S810490*, *Aannua14947S778390*, *Aannua05209S490740* and *Aannua02702S329990* in AaAPK1-RNAi transgenic *Artemisia* and wild type *Artemisia*. WT: wild-type *A. annua* plants.
